# Supplementary figures and images for: Harmonic radar tracking of individual melon flies, Zeugodacus cucurbitae, in Hawaii: Determining movement parameters in cage and field settings
Source: PLoS One. 2022 Nov 16;17(11):e0276987. doi: 10.1371/journal.pone.0276987 (PMC9668202; doi:10.1371/journal.pone.0276987)

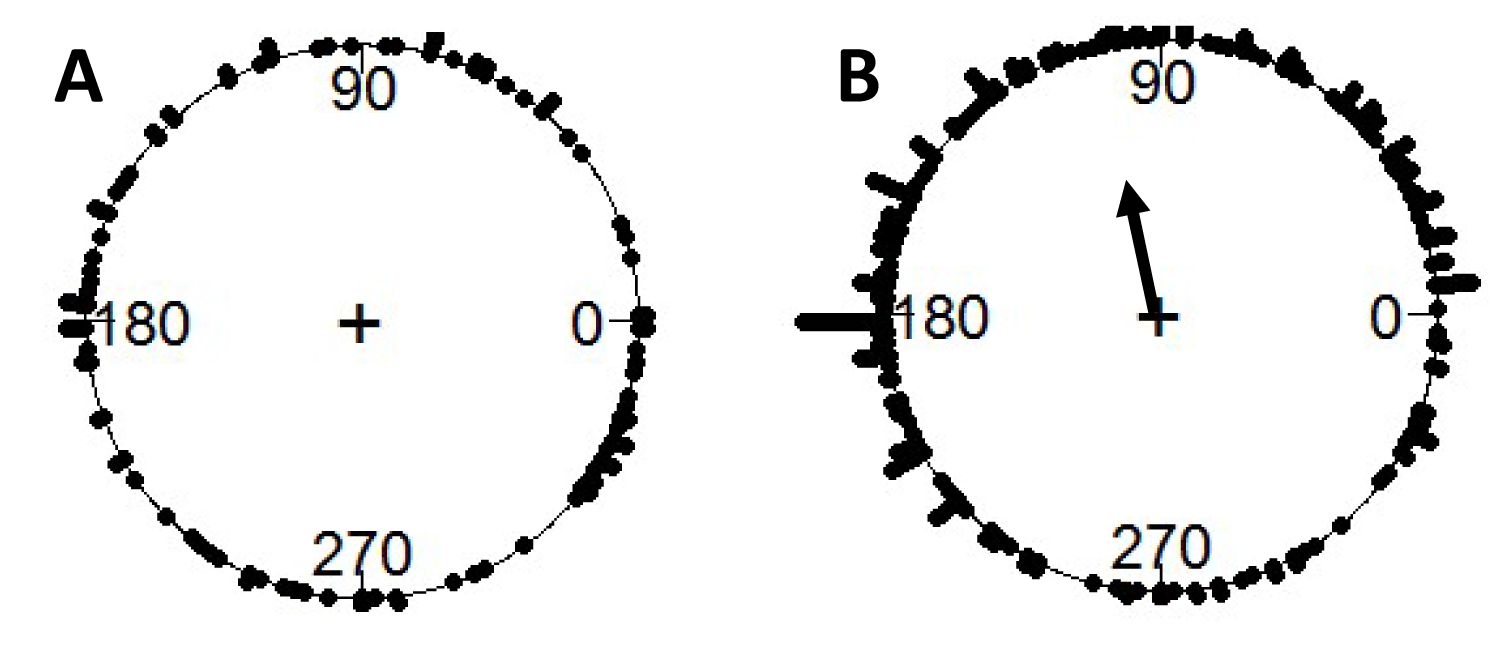

Supplement: S1 Fig — Combined fly flight directions for experiments 2 (A) and 3 (B). Black arrow: wind direction; +: Release point. Flight directions were homogeneous for experiment 2 (P = 0.9433, Rayleigh test; P = 0.2160, Hermans-Rasson test) while experiment 3 flight directions were not homogeneous but showed directionality (P < 0.001, Rayleigh test; P < 0.001, Hermans-Rasson test). A V-test for experiment 3 showed a unimodal distribution correlated with the mean wind direction (P < 0.001). (TIF) [file pone.0276987.s001.tif]

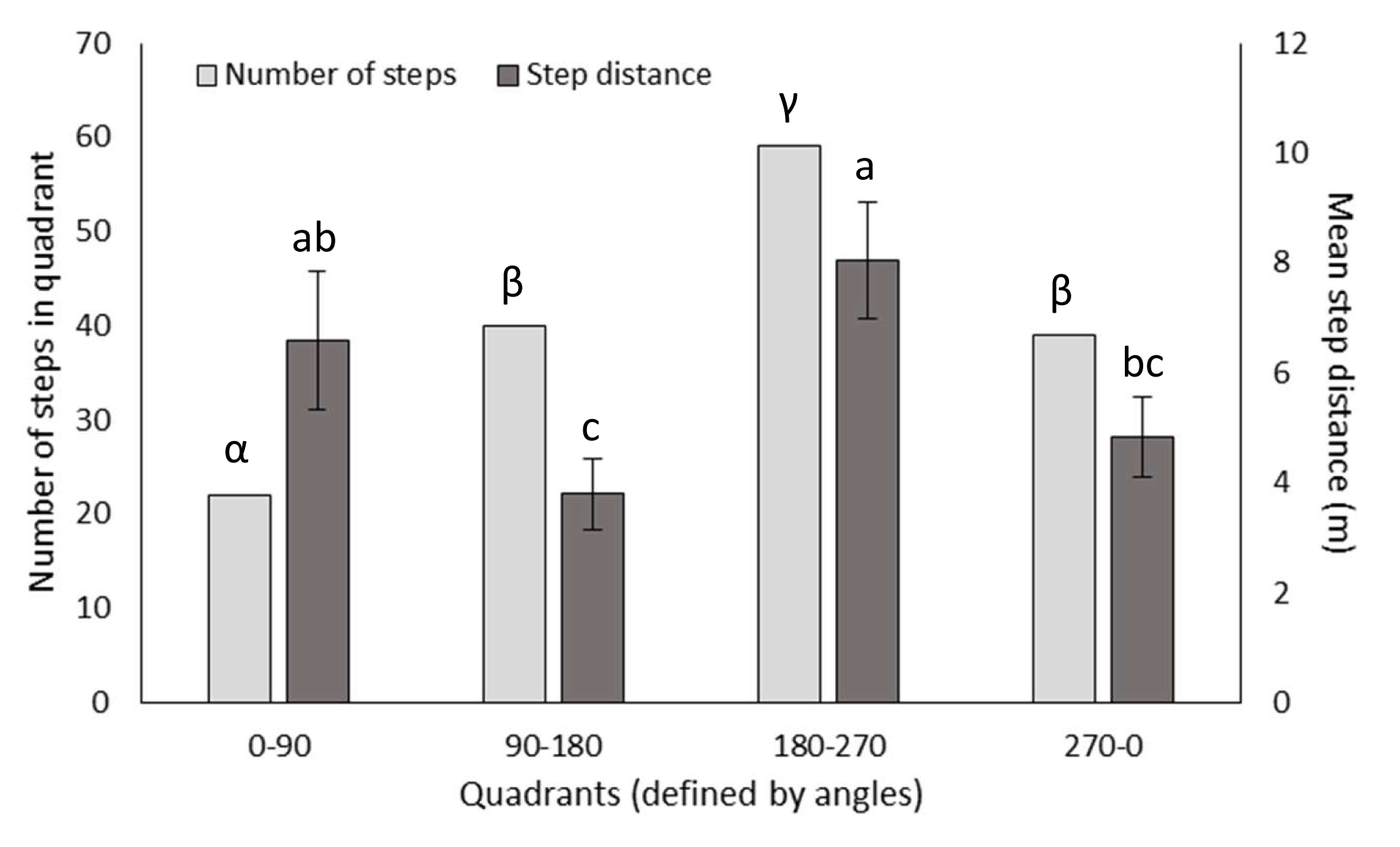

Supplement: S2 Fig — For steps in quadrant 1, flies flew against the wind. For steps in quadrant 3, flies flew with the wind. For steps in quadrants 2 and 4, flies flew across the wind. Using a contingency table approach, the number of steps per quadrant were found to be unequal (χ2exp (22.867) > χ2crit (16.266), df = 3, P < 0.001). Quadrants marked by different Greek letter have significantly different proportions of steps (Marascuillo procedure). Mean step distances with different Arabic letters are significantly different between quadrants (ANOVA, Tukey’s HSD). (TIF) [file pone.0276987.s002.tif]

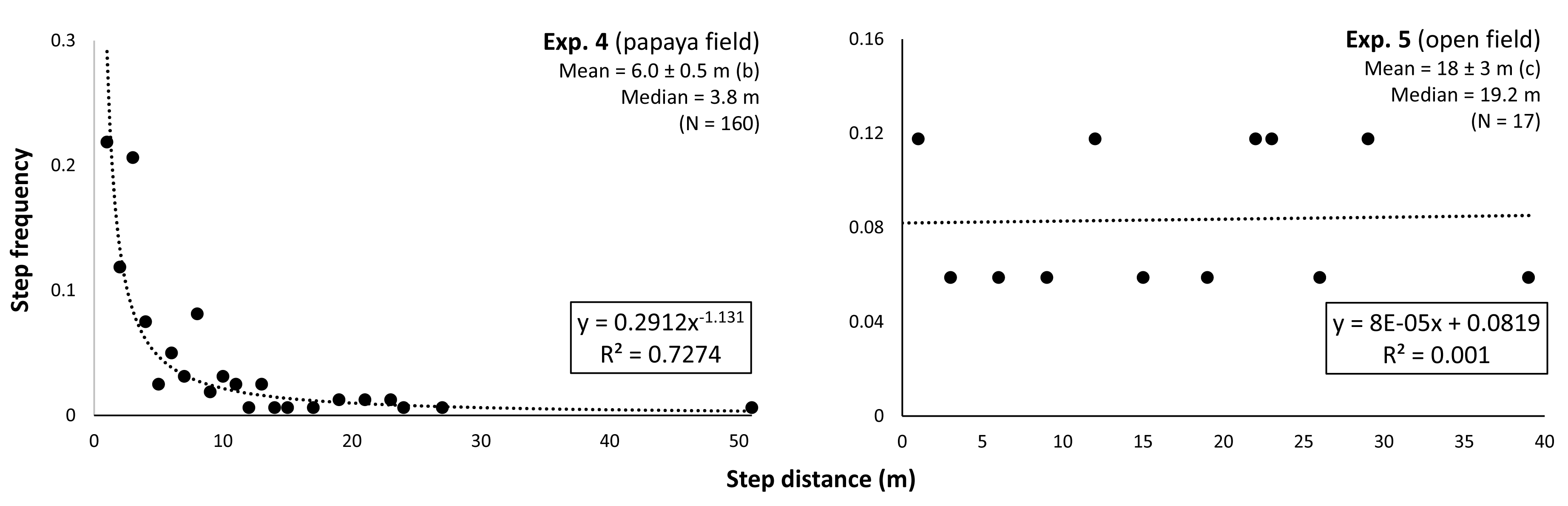

Supplement: S3 Fig — For experiment 4, step-distances of less than 1 m were removed. (TIF) [file pone.0276987.s003.tif]
