# Supplementary material for: Harmonic radar tracking of individual melon flies, Zeugodacus cucurbitae, in Hawaii: Determining movement parameters in cage and field settings
Source: PLoS One. 2022 Nov 16;17(11):e0276987. doi: 10.1371/journal.pone.0276987 (PMC9668202; doi:10.1371/journal.pone.0276987)
Supplement: Attachment — Submitted filename: Response to Reviewers.docx [file pone.0276987.s006.docx]

**Responses to specific comments:**

**Associate Editor**

1. Please ensure that your manuscript meets PLOS ONE's style requirements, including those for file naming.

- We have re-checked and believe we are meeting all of PLOS ONE's style requirements.

2. In your Methods section, please provide additional information regarding the permits you obtained for the work. Please ensure you have included the full name of the authority that approved the field site access and, if no permits were required, a brief statement explaining why.

- The following text has been added to both the descriptions of the papaya and open field experiments, “No permit was required for access to this field site.”

3. We note that the grant information you provided in the ‘Funding Information’ and ‘Financial Disclosure’ sections do not match. When you resubmit, please ensure that you provide the correct grant numbers for the awards you received for your study in the ‘Funding Information’ section.

- We have resolved the mismatch.

4. Thank you for stating the following in the Acknowledgments Section of your manuscript…

- Funding information has been removed from the Acknowledgement. We would like our Funding Statement to read as follows,

“"NCM, MSS, LAFN - This study was supported in part by ARS project 2040-22430-027-00D, United States Department of Agriculture, Agricultural Research Service, www.ars.usda.gov/

NDM, TJY - This study was supported in part by a Kauffman and Miller Research Award, Daniel B. Suter Endowment in Biology, Eastern Mennonite University, emu.edu/science-seminars/daniel-b-suter-endowment

There was no additional external funding received for this study.

The funders had no role in study design, data collection and analysis, decision to publish, or preparation of the manuscript."

There is no award number for the Kauffman and Miller Research Award.

5. We note that Figure 9 in your submission contain satellite image which may be copyrighted…

- The Figure 9 has been replaced with a map that we independently generated that does not include any copyrighted material. The following text has been added to the Figure 9 legend, “Source: U.S. Geological Survey, 2011, USGS High Resolution State Orthoimagery for the East Side of Hawaii Island: 05QKB820780_200912_0x5000m_CL_1: U.S. Geological Survey.”

**Reviewer 1**

*General comments*

Firstly, please discuss only results relevant to male flies, you did not release female flies as far as I could tell. Therefore, remove any references to oviposition etc...

- The sentence in the discussion that mentions oviposition has been rewritten. We have examined the discussion to ensure that we do not discuss any female specific behaviors as the reviewer has rightly pointed out that we did not track females in this study.

I strongly suggest to reduce the Discussion, it is much too long… Secondly, please reduce the discussion by removing unnecessarily lengthy comparisons with other literature. For example, discussion on wind direction: State that the following references found flies did orientate with wind direction, while the following did not. You do not need to explain what each reference found in detail.

- The discussion has been edited to remove multiple lengthy literature comparisons. As the reviewer suggested, we have condensed sections of text in an attempt to avoid discussing each reference in extended depth. Specific sections that were shorten include passages addressing literature on wind direction, flight distances, and comparisons of HR tracking with other ways fly movement might be studied. These reductions have resulted in the removal of approximately one and a half pages of text.

I would also give the whole document another detailed read to remove spelling and grammatical errors, I am not sure I caught them all.

- The entire manuscript has been read over again with several additional errors corrected.

*Specific comments*

Line 58 – corrected as suggested

Line 59 – corrected as suggested

Line 61 – corrected as suggested

Line 70 – corrected as suggested

Line 71 – trapping networks are broader than just Medfly

Line 97 – corrected as suggested

Line 106 – corrected as suggested

Line 108 – corrected as suggested

Line 109 – corrected as suggested

Line 141 – info added as suggested

Line 170 – corrected as suggested

Lines 202-203 – text added to clarify as suggested

Line 206 – text added as suggested

Line 225 – text added as suggested

Line 237 – text added as suggested

Line 279 – corrected as suggested

Line 323 – corrected as suggested

Line 332 – corrected as suggested

Line 347 – corrected as suggested

Lines 358-359 – sentence removed as suggested

Line 385 – corrected as suggested

Line 427 – corrected as suggested

Line 393 – corrected as suggested

Line 401 – corrected as suggested

Line 405 – corrected as suggested

Line 454 – changed to “short-range”

Line 471 – corrected as suggested

Line 474 – corrected as suggested

Line 509 – corrected as suggested

Line 512 – corrected as suggested and sentence removed as suggested

Line 518 – corrected as suggested

Line 520 – corrected as suggested

Line 522 – CRW is defined earlier in the paper

Line 551 – added full name as suggested

Line 555 – added full name as suggested

Line 559 – added full name as suggested

Lines 561-563, 575-578, 586-588, 605-609 – We are not familiar with a scientific writing style guide that forbids results in figure legends. In fact, we found several style guides that encourage the inclusion of brief results and reporting statistics in figure legends. We believe the legends are not overly long and would enhance the readers understanding of the figure. If the editor feels we should remove these we will do so.

Line 565 – added full name as suggested

Line 569 – added full name as suggested

Line 573 – added full name as suggested

Line 580 – added full name as suggested

Line 584 – added full name as suggested

Line 590 – added full name as suggested

Line 611 – added full name as suggested
